# Case Report: Treatment of hypersomatotropism in a diabetic dog with transsphenoidal hypophysectomy

**DOI:** 10.3389/fvets.2026.1740713

**Published:** 2026-02-24

**Authors:** Anika S. Meij, Lucinda Luvia Van Stee, Hedwig S. Kruitwagen, Robert Cornelis van Nieuwaal-Jubbega, Guy C. M. Grinwis, Sara Galac, Björn P. Meij

**Affiliations:** 1 Animal Referral Centre, Auckland, New Zealand; 2 Department of Clinical Sciences, Faculty of Veterinary Medicine, Utrecht University, Utrecht, Netherlands; 3 Department of Pathobiology, Faculty of Veterinary Medicine, Utrecht University, Utrecht, Netherlands; 4 Department of Biomolecular Health Sciences, Faculty of Veterinary Medicine, Utrecht University, Utrecht, Netherlands

**Keywords:** acromegaly, diabetes mellitus, growth hormone, pituitary adenoma, veterinary neurosurgery

## Abstract

**Background:**

Pituitary somatotroph adenoma is rare in dogs and may cause hypersomatotropism (HS) leading to insulin resistance and diabetes mellitus (DM).

**Case description:**

A 10-year-5-month-old neutered male Staffordshire Bull Terrier presented with polyuria, polydipsia, progressive inspiratory stridor, and poorly controlled DM with hyperinsulinemia and insulin resistance. Serum insulin-like growth factor (IGF-1) was markedly elevated (1,214 ng/mL; reference interval, 42–449 ng/mL) and suggested HS which was further supported by a somatostatin suppression test. Magnetic resonance and computed tomography (CT) imaging revealed a pituitary mass, organomegaly, and arthropathy.

**Treatment and outcome:**

The pituitary mass was removed by transsphenoidal hypophysectomy. Immunohistochemistry confirmed a growth hormone (GH)-producing pituitary adenoma. Postoperatively, GH normalized within hours, and IGF-1 values within a week. Although HS resolved and hyperinsulinemia improved postoperatively, the dog remained dependent on insulin and DM persisted which eventually led to euthanasia of the dog about 9 and a half months post-operatively.

**Conclusion:**

Transsphenoidal hypophysectomy was effective in normalization of GH and IGF-1 concentrations in a dog diagnosed with a pituitary somatotroph adenoma but the postoperative course was characterized by persistent insulin dependency and DM.

## 1 Introduction

Chronic hypersecretion of GH by a functional pituitary somatotroph adenoma causes HS. Excess secretion of GH and subsequent increased insulin-like growth factor-1 (IGF-1) may lead to physical changes known as acromegaly. These changes include soft tissue overgrowth (like excessive skin folds and macroglossia) and skeletal overgrowth (like widened interdental spaces). Most cases also develop insulin-resistant DM. The pituitary somatotroph adenoma is rare in dogs but represents the most common pituitary tumor in cats (1). Transsphenoidal hypophysectomy has been described as a successful treatment for somatotroph pituitary tumors in cats (2, 3).

This case report describes the diagnosis, management, and outcome of a dog with HS and DM associated with a pituitary somatotroph adenoma that was treated by transsphenoidal hypophysectomy.

## 2 Case description

**Figure 1:**
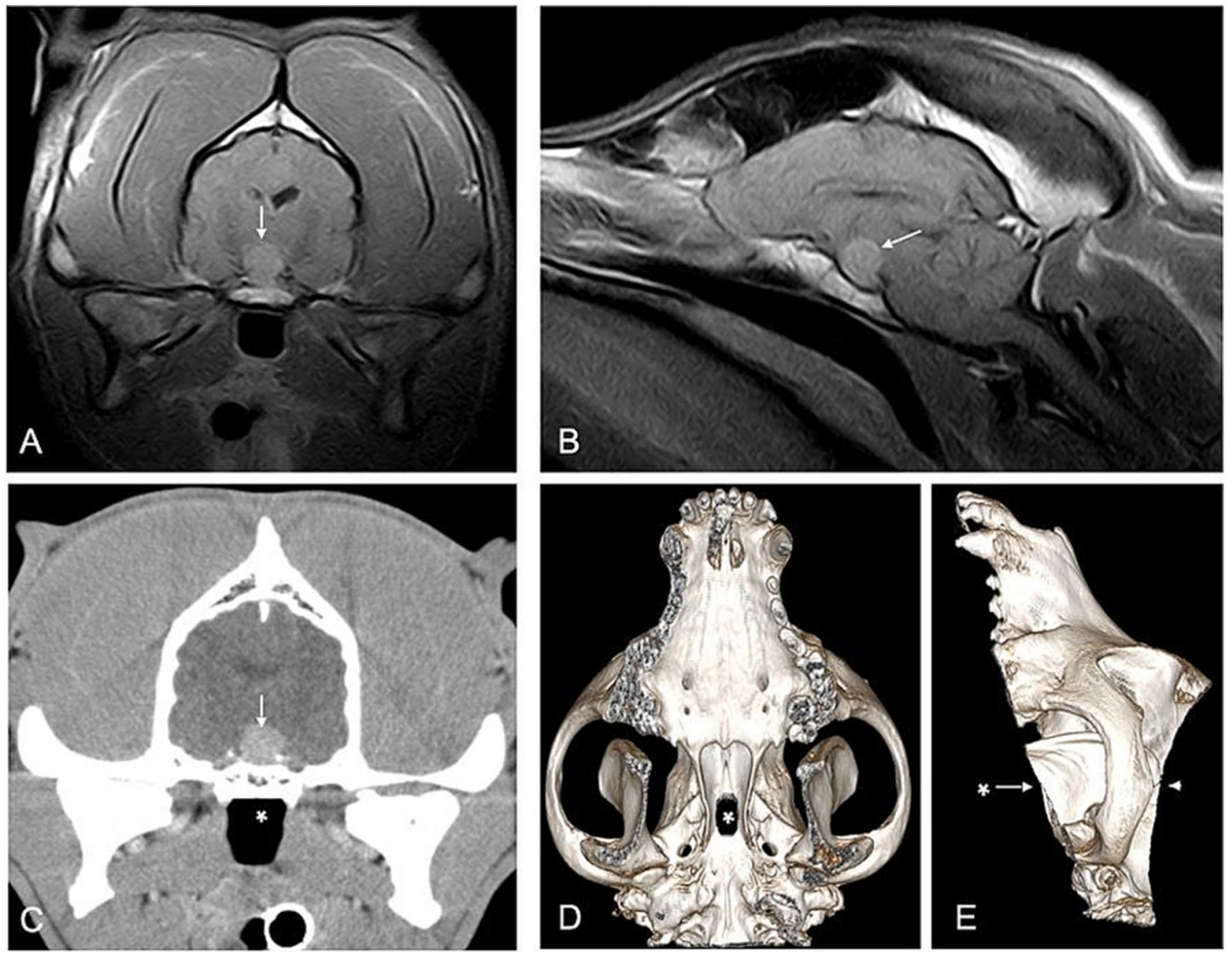
Imaging and planning for hypophysectomy in a 10-year-old Staffordshire Bull Terrier with pituitary somatotroph adenoma. Coronal **(A)** and sagittal **(B)** MRI and contrast-enhanced CT **(C)** showed an enlarged pituitary mass (arrow) measuring 12 × 10 mm with a pituitary height/brain area value of 0.69. Planning of hypophysectomy was performed on a 3D CT skull reconstruction **(D,E)** and *in silico* localization of the burr hole (*) in the sphenoid bone to exit precisely at the pituitary fossa which was confirmed inside the calvarium (arrowhead).

A 10 year-5 month-old male neutered Staffordshire Bull Terrier weighing 39.3 kg, was presented with polyuria and polydipsia, weight loss, exercise intolerance and progressive inspiratory stridor (Supplementary Figure S1). On physical examination, the dog showed gingival hyperplasia and widened interdental spaces with no other physical abnormalities. Hematology and serum biochemistry revealed mild thrombocytosis, hyperglycemia of 30 mmol/L [reference interval (RI), 4.1–7.95 mmol/L], elevated fructosamine of 658 μmol/L [RI 194–399 μmol/L] and mildly elevated cholesterol. Total plasma thyroxine (T4) and thyroid-stimulating hormone (TSH) concentrations were in the normal range, respectively 25.8 nmol/L [RI, 6.5–43.9 nmol/L] and 0.12 ng/mL [RI, <0.5 ng/mL]. Urinalysis revealed glucosuria with a specific gravity of 1.031. The dog was diagnosed with DM and treatment was initiated with subcutaneous injection of lente insulin (Caninsulin, MSD Animal Health Boxmeer, Netherlands) at 0.5 IE/kg twice daily. Due to poor glycemic control and persistent clinical signs of DM, the insulin dose was increased several times over a period of 1 month. HS was suspected by measurement of serum IGF-1 concentration of >1,000 ng/mL [RI, 42–449 ng/mL]. The dog had no history of treatment with progestins. Ultrasound of the abdomen showed no significant abnormalities. Magnetic resonance imaging (MRI) of the neurocranium revealed a subjective flattening of the sulci and gyri of the cerebrum. Asymmetry of the lateral ventricles was visible. A well-defined T1- and T2- isointense pituitary mass of 13 × 11 mm was visible with extension to the dorsal suprasellar region (Figures 1A,B). There was mild mass effect on the thalamic region. No other visible abnormalities were present on the images.

**Figure 2:**
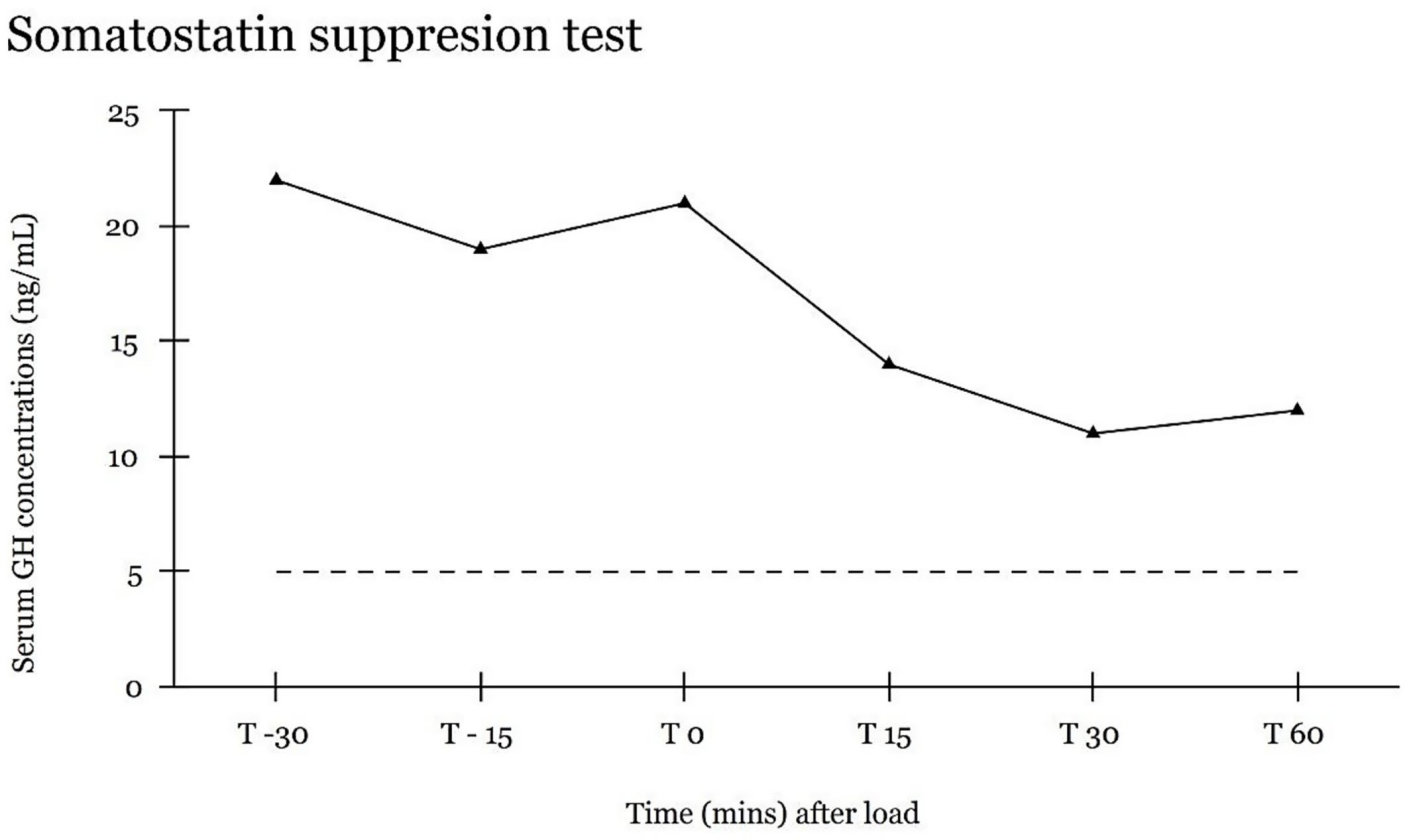
Somatostatin suppression test. Serum GH concentrations in blood samples collected at 30 and 15 min before, and at 15, 30, and 60 min after intravenous administration of 10 μg somatostatin/kg body weight. Dashed line indicates upper level of RI [2–5 ng/mL] of plasma GH (25).

The dog was referred for treatment to the Department Clinical Sciences of the Faculty of Veterinary Medicine of the Utrecht University, Netherlands. At the time of referral, the dog was treated with 2 IE/kg (80 IU) of lente insulin twice daily. However, the regulation of DM was unsatisfactory suggesting insulin resistance. Blood glucose measured 31.7 mmol/L [RI, 4.2–5.8 mmol/L] and fructosamine 678.0 μmol/L [RI, 189–340 μmol/L]. For the convenience of monitoring daily variations in glucose, a glucose monitoring device (Freestyle Libre, Abbott, United States) was placed subcutaneously in the neck region of the dog. The blood glucose day curve demonstrated persistently extreme hyperglycemia, with the lowest measured plasma glucose concentration of 22 mmol/L occurring 6 h post-insulin administration. Receiving 2 IE/kg of lente insulin, these findings were consistent with insulin resistance. Repeated measurement of serum IGF-1 was elevated [1,214 ng/mL; RI, 42–449 ng/mL] (4). Hypercortisolism was excluded by two normal urinary corticoid:creatinine ratios [5.2 and 3.0 × 10^−6^ (RI < 10 × 10^−6^)] and normal suppression of serum cortisol in a low dose dexamethasone suppression test. Serum basal cortisol concentrations at 0, 4, and 8 h post dexamethasone were, respectively, 48, 10, and 8 nmol/L, with the 8 h cut off <40 nmol/L (5). Plasma insulin concentration was elevated at >300 mU/L [RI, <20 mU/L] (5). A somatostatin suppression test was performed, by administering 10 μg/kg of somatostatin (Somatostatin-UCB, UCB Pharma B.V., Netherlands) intravenously and GH values were measured up to 60 min afterwards (Figure 2). The plasma GH values did not decrease below 10 ng/mL [RI 2–5 ng/mL] (6, 7).

CT was performed, and contrast enhancement revealed a pituitary mass of 11.7 mm (height) × 9.6 mm (width) × 9.4 mm (length) with a brain area of 16.89 cm^2^ (Figure 1C). The pituitary height/brain area value (*P*/*B*) was 0.69 [RI, <0.31 for non-enlarged pituitary (8)]. Other abnormalities included hepatomegaly, marked bilateral elbow joint osteoarthritis, most likely secondary to fragmented medial coronoid processes, mild bilateral osteoarthritis of the shoulder joints, ventral spondylosis of the thoracic and lumbosacral spine, ossifying pachymeningitis of the cervical and thoracic spine, and mineralization of the right caudal oblique capital muscle.

Transsphenoidal hypophysectomy was performed according to the technique previously described (9, 10). Surgical localization of the burr hole was prepared on a 3D reconstruction of the CT scan (Figures 1D,E). Briefly, following incision and retraction of a thickened soft palate, it was noted that the mucoperiosteum was thicker than normally experienced in cushingoid dogs, and covered the sphenoid bone in thick folds. Access to the pituitary fossa was obtained through the 10 mm thick sphenoid bone with an electrically powered burr and Love Kerrison rongeurs were used to enlarge the sphenoid bone burr slot. The dura mater was coagulated and incised, and a stark white pituitary adenoma was detached from the pituitary fossa using a small ball-tipped probe and cup forceps. Hypophysectomy was assessed to be complete by visualization of the entrance to the third ventricle and an empty fossa. The surgical pituitary specimen was sent for histopathological examination. Closure was performed routinely, and the dog was hospitalized in the intensive care unit.

Recovery from surgery was uncomplicated; the dog started to eat and drink on the second day after surgery. Perioperatively glucose was controlled with a continuous rate infusion of insulin lispro (Humalog, Eli Lilly, Utrecht, Netherlands). Postoperatively managing glycemic values was challenging. Insulin lente was started on 0.3 IE/kg (13 IU) every 12 h, but due to insufficient control of hyperglycemia the dose was increased to 1.8 IE/kg (70 IU) every 12 h in 7 days (Supplementary Figure S2). Glucose control remained unsatisfactory, and lente insulin was changed to protamine zinc insulin (ProZinc, Boehringer Ingelheim, Amsterdam, Netherlands). This resulted in variable glycemic control, with periods of hyperglycemia and hypoglycemia. Protamine zinc insulin appeared to have an unexpected, prolonged duration of action, with the lowest blood glucose concentrations measured 17 to 21 h after administration. Consequently, therapy was switched to a shorter-acting human insulin (Actrapid, Novo Nordisk A/S, Bagsværd, Denmark). A dose of 0.5 IU/kg (20 IU) three times daily was started 20 days postoperatively. Glucose levels stabilized alongside a feeding regime of 175 g per meal with every insulin dose. The dog was discharged after 24 days of hospital care. Medication after surgery consisted of one drop (5 μg) desmopressin (Minrin, Ferring, Hoofddorp, Netherlands) three times daily in the conjunctival sac for 3 weeks, 15 mg cortisone acetate (Cortisonacetaat, Alfasan B.V., Netherlands) three times daily lifelong, and 600 mg thyroxine (Forthyron, Dechra Veterinary Products, Shrewsbury, United Kingdom) every 12 h lifelong according to the routine postoperative protocol (11).

**Figure 3:**
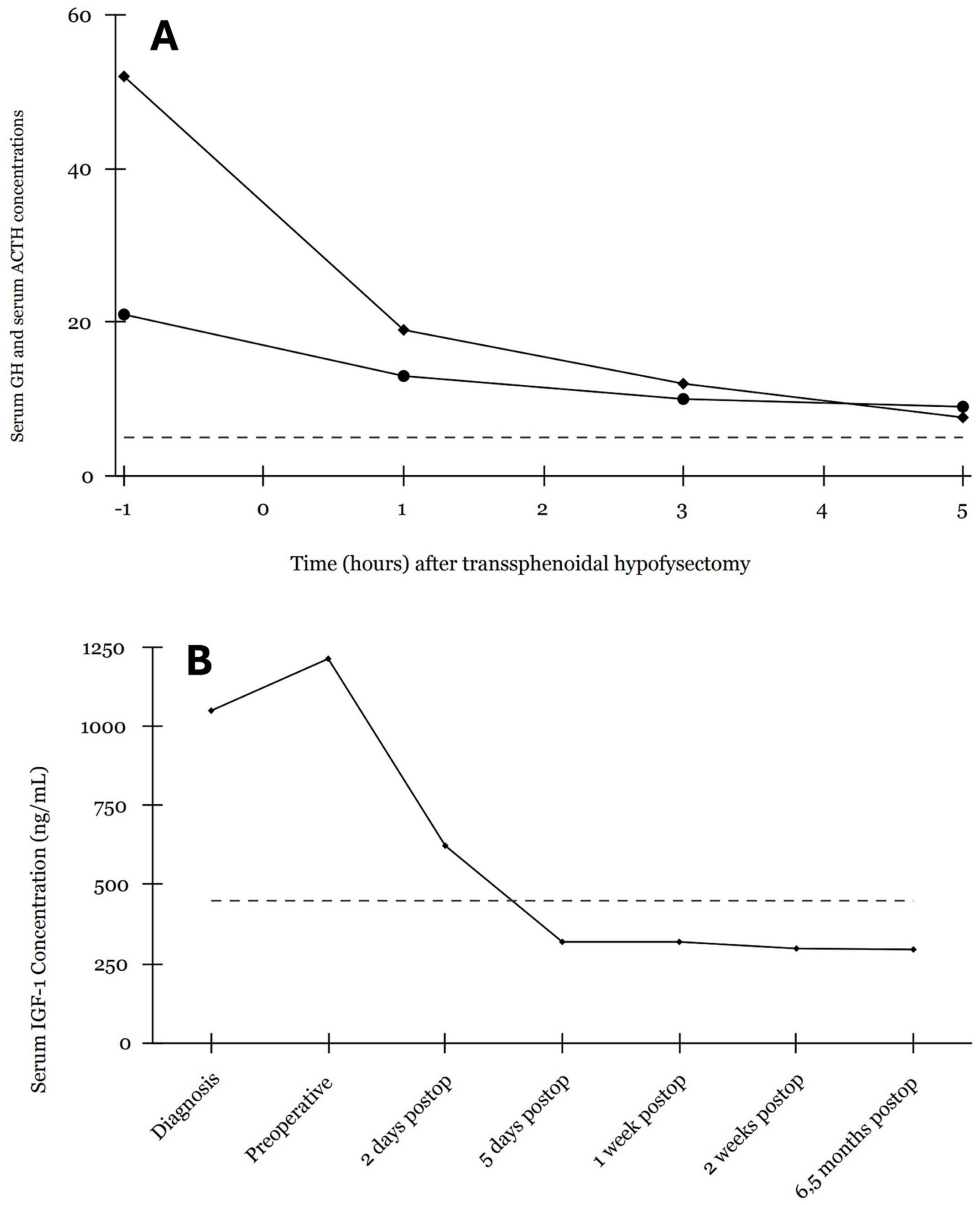
Peri- and post-operative changes in plasma GH in ng/mL, adrenocorticotropin (ATCH) in pg/mL and serum IGF-1 in ng/mL. **(A)** GH (♦) and ACTH (•) concentrations before and at 1, 3, and 5 h after hypophysectomy in a diabetic dog with HS. The dashed line indicates the upper level of RI [2–5 ng/mL] of plasma GH (25). **(B)** IGF-1 concentrations before and after hypophysectomy in a dog with HS. The dashed line indicates the upper limit of the RI for IGF-1 in healthy dogs [RI 42–449 ng/mL] (4).

Perioperative plasma GH and adrenocorticotropic hormone (ACTH) concentrations were measured before and, respectively, at 1, 3, and 5 h after transsphenoidal hypophysectomy. Elevated plasma GH concentrations rapidly decreased from 52 ng/mL to near normal level of 7.6 ng/mL at 5 h post-hypophysectomy (Figure 3A). Basal plasma ACTH, with values at two separate times of 15 and 21 pg/mL [RI < 60 pg/mL], were reported to be normal preoperatively (9). Postoperatively the ACTH values decreased further to 9 pg/mL at 5 h after hypophysectomy (Figure 3A). The serum IGF-1 concentration normalized rapidly in the first week postoperatively (Figure 3B). At 1 week postoperatively, plasma insulin concentration had decreased to 42 mU/L [RI, <20 mU/L] (4).

**Figure 4:**
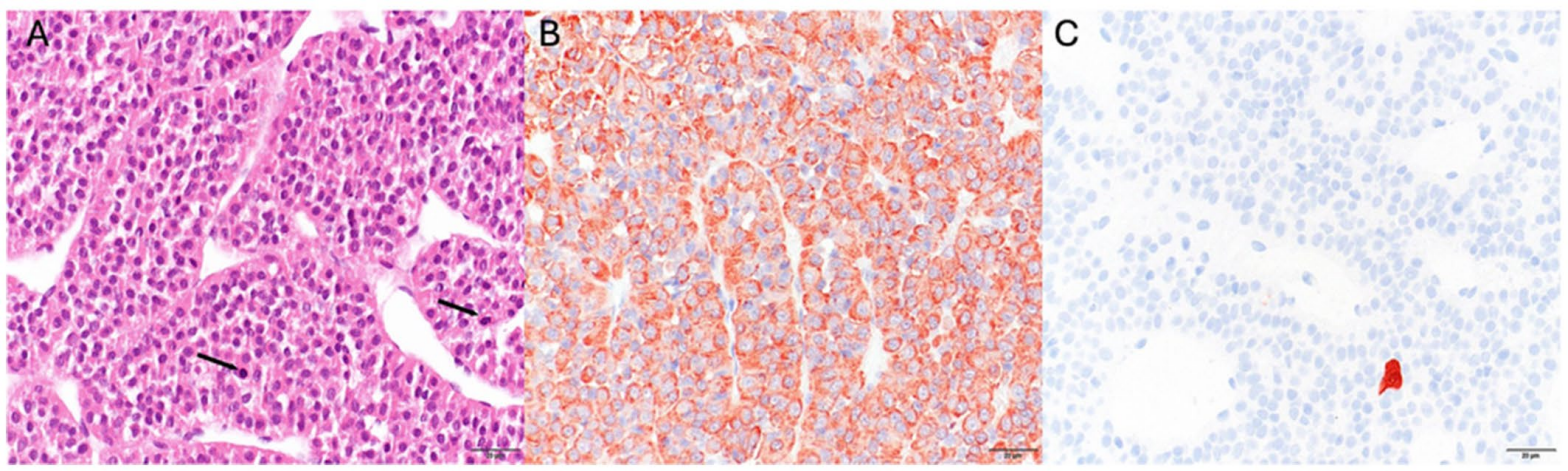
**(A)** Histology of the acidophilic adenoma. Neoplastic cells are grouped in trabecula with intertrabecular thin-walled blood vessels. Two mitotic figures are visible (arrows; hematoxylin and eosin (H&E) stain). **(B)** Neoplastic cells show granular, cytoplasmic immunoreactivity for GH. **(C)** A single, preexisting, corticotropic cell shows marked cytoplasmic immunoreactivity for ACTH, the neoplastic cells are negative. All images objective 40×.

Histopathological examination of the pituitary tissue demonstrated a neoplastic proliferation composed predominantly of acidophilic, polygonal cells arranged in anastomosing trabeculae around optically clear vascular spaces, supported by minimal stromal tissue. The tumor cells displayed abundant eosinophilic, granular cytoplasm (Figure 4A), mild anisocytosis, and centrally to basally located nuclei with fine granular chromatin and one to two small nucleoli and few mitotic figures (3 in 2.37 mm^2^). Immunohistochemical staining was performed by the avidin-biotin technique using a monoclonal mouse antibody to synthetic adrenocorticotrophic hormone (ACTH; 1–24) (Department of Infectious Diseases and Immunology, Faculty of Veterinary Medicine, Utrecht University, Netherlands), and a polyclonal rabbit antibody to porcine GH (source 4,750–3,959, Biogenesis, Poole, United Kingdom) (12). Immunohistochemical staining demonstrated strong cytoplasmic staining for GH in the majority of tumor cells (Figure 4B). No immunoreactivity for ACTH was noted in the neoplastic cells (Figure 4C). These findings were consistent with a GH producing eosinophilic pituitary adenoma.

At 6.5 months postoperatively, IGF-1 plasma concentration was 295 ng/mL confirming continued remission of HS (Figure 3B). Clinically, the dog was reported to have gained significant weight (15 kg over 2 months), showed less energy and reduced mobility, with persistent hunger at this time. Urination was unremarkable, though slightly increased drinking was suspected. No information was available regarding remission of the inspiratory stridor or the widened interdental spaces. Despite administration of 0.3 IE/kg (16 IU) three times daily of insulin (Actrapid, Novo Nordisk A/S, Denmark), fructosamine level of 535 μmol/L [RI, 189–340 μmol/L] revealed poorly controlled DM. T4 levels were measured at 36 nmol/L at this time to rule out underlying hypothyroidism [RI, 6.5–43.9 nmol/L].

At 286 days after transsphenoidal hypophysectomy the dog was euthanized because of poor control of DM and difficulty for the owners to keep the dog at a comfortable level. No necropsy was performed.

## 3 Discussion

This case report describes the clinical presentation of a dog with HS due to pituitary somatotroph adenoma. Absence of previous treatment with progestins excluded a mammary origin of GH production (13). The dog was presented with features of acromegaly and insulin resistant uncontrollable DM. The diagnosis of HS in this dog was confirmed by a markedly elevated (IGF-1) concentration of 1,214 ng/mL [RI, <1,000 ng/mL] (14, 15). Advanced imaging with MRI and CT revealed evidence of a pituitary mass and histology confirmed a GH producing adenoma. Treatment by transsphenoidal hypophysectomy resulted in remission of HS, as evidenced by normalization of IGF-1 within 1 week postoperatively up to 6.5 months postoperatively. However, the DM persisted and although the glycemic control improved, the management was not optimal. Difficulty to cope with the diabetic situation led to euthanasia at 286 days post-surgery.

Dogs suffering from DM can have concurrent disorders that cause insulin resistance because of their specific pathophysiology. The most common concurrent disorders in diabetic dogs include hypercortisolism, urinary tract infection, acute pancreatitis, neoplasia and hypothyroidism (16, 17). While in cats, HS is the most common differential in insulin-resistant DM, in dogs it remains rare. A comprehensive diagnostic work-up was performed in the dog of the present study to identify the cause of marked insulin resistance and to exclude other potential contributors. Normocortisolism was confirmed through two basal urine corticoid-to-creatinine ratios. Also, a normal low-dose dexamethasone suppression test effectively ruled out hypercortisolism (18). A recent case report showed that concurrent HS and hypercortisolism can occur in the same dog caused by a plurihormonal pituitary adenoma, secreting both excess GH and ACTH (4). Abdominal ultrasonography and further biochemistry bloodwork revealed no abnormalities suggestive of pancreatitis or other neoplasia. Although glucosuria was present, no evidence of urinary tract infection was found. Basal total T4 and TSH concentrations were within RIs, making hypothyroidism unlikely (19). Ruling out hypothyroidism is also important because it is associated with elevated GH release and IGF-1 levels (20).

Both elevated GH and IGF-1 levels play a central role in the pathophysiology of the acromegalic dog with insulin resistant DM. The effects of circulating GH can be divided into two main categories: rapid catabolic actions and slow (long-lasting) hypertrophic actions. The acute catabolic actions are mainly due to insulin antagonism and result in enhanced lipolysis, gluconeogenesis, and restricted glucose transport across the cell membrane. The net effect of these catabolic actions is promotion of hyperglycemia (7, 21, 22). The GH anti-insulin effect is the main cause of insulin resistance (21, 22). The slow anabolic effects are mainly mediated via IGF-1. IGF-1 is produced in many different tissues and, in most of these tissues, has a local growth-promoting effect on cartilage, bone, and other tissues. The main source of circulating IGF-I is the liver, and its synthesis is promoted by GH. Elevated IGF-1 levels reflect high circulating GH levels. Therefore, elevated GH levels causing hyperglycemia, insulin resistance, and DM go hand in hand with elevated IGF-1 levels (HS). Local growth-promoting effects through IGF-1 on organs can lead to organ enlargement, e.g., the heart, resulting in hypertrophic cardiomyopathy in cats with HS (23), but also mucosal thickening in the mouth (tongue, gingiva, nasal passage) resulting in inspiratory stridor. Indeed, increased thickness of the soft palate, thick folds of mucoperiosteum and hepatomegaly were found in the dog in the present study.

Excess of GH (and IGF-1) can also have a long-lasting growth promoting effect on the pancreatic beta cells, leading to increased endogenous insulin production. Hyperinsulinemia like in the present case [>300 mU/L; RI, <20 mU/L] with administration of 2 IU/kg insulin lente BID and persistent hyperglycemia suggested insulin resistance. The highly elevated serum insulin concentration decreased to near normal values at 1 week after hypophysectomy [42 mU/L; RI, <20 mU/L] showing that restoration of GH levels to basal levels, as recorded in our case within hours after hypophysectomy, can effectively return the beta cells to almost normal insulin production. However, the half-life of GH (hours) is much shorter than that of IGF-1, and therefore normalization of GH concentrations is expected within days after complete hypophysectomy, whereas IGF-1 levels typically normalize only after several weeks. Persistent hyperglycemia in our case may be explained by ongoing insulin resistance in peripheral tissues, even when IGF-1 concentrations had returned to normal.

A somatostatin suppression test was performed to demonstrate the lack of suppression of serum GH concentrations. In healthy dogs, somatostatin effectively inhibits GH secretion through negative feedback mechanisms (24). In the present case, GH concentrations remained above 10 ng/mL following somatostatin administration. For this patient, lack of suppression of elevated plasma GH levels by somatostatin further supported the diagnosis of HS (7). In normal healthy dogs, GH values are expected to decline within the normal range (2–5 ng/mL) following somatostatin administration (6). The minimal decline of plasma GH levels to suppression by somatostatin further supported the diagnosis of HS (7). Similar findings have been reported in previous studies where somatostatin failed to suppress GH to normal levels in two dogs with HS due to a pituitary tumor (6, 25), and in a cat with HS (26).

Pituitary histopathology of dogs with HS has rarely been described (25, 27, 28). Two cases were diagnosed with somatotroph adenoma based on postmortem pathologic examination (25, 28). Following a previous report by Steele et al. (27) this is the second report of a dog with a pituitary somatotroph adenoma treated by transsphenoidal hypophysectomy. Definitive diagnosis of HS was achieved through increased serum IGF-1 concentration and advanced imaging plus pituitary histology, similar to the previous case (27). Similarities include some clinical signs, like the inspiratory stridor and polyuria and polydipsia. But the previous case with a presentation of acromegaly (27) was initially submitted without a clear diagnosis of DM and the dog only developed hyperglycemia just prior to surgery, while our case was admitted with uncontrollable DM. In both cases, HS went into remission, but DM persisted postoperatively. Cats with HS on the other hand, have shown good response regarding remission of DM after hypophysectomy. In the study by van Bokhorst et al. (2), 22 of 24 cats (92%) showed remission of DM after hypophysectomy, and 41 of 68 cats (60.3%) in the study by Fenn et al. (3). Feline pancreatic *β*-cells appear to retain a notable capacity for functional recovery, even following prolonged periods of poor glycemic control despite administration of high doses of exogenous insulin (2). In contrast, dogs generally remain insulin dependent (29). As reported in the literature, dogs with DM secondary to hypercortisolism typically exhibit irreversible β-cell dysfunction, and insulin dependence persists despite management of the underlying condition (29). It appears that in the dog with endocrine active pituitary adenomas and concurrent DM, unlike in the cat, reversibility of resistance to insulin in peripheral tissues is not complete, which explains persistent DM after hypophysectomy.

The authors hypothesize that this difference may be related to species-specific genetic factors combined with their differing nutritional backgrounds (mixed omnivore/carnivore in dogs versus strict carnivore in cats). Therefore, remission of HS is a valuable prognostic parameter for complete hypophysectomy in the dog but does not necessarily mean that DM will be reversed.

In the present case, a marked weight gain (15 kg) accompanied by persistent polyphagia was observed. It remains unclear to what extent this was a direct consequence of HS, the postoperative glucocorticoid replacement therapy, or an additional endocrinopathy such as (iatrogenic) hypercortisolism or hypothyroidism, which in retrospect might have warranted further investigation. This vicious cycle of polyphagia, obesity, and peripheral insulin resistance can complicate diabetes management and additionally contributes to musculoskeletal strain and reduced quality of life. In human medicine, novel pharmacological interventions such as GLP-1 receptor agonists are being used to reduce appetite and limit weight gain; similar strategies may hold promise for companion animals in the future (30). Further studies are required to better elucidate the pathophysiology of weight gain in dogs with HS and to determine which therapeutic approaches are effective and feasible in veterinary practice.

Our case appears unique due to the specific pattern of skeletal abnormalities like bilateral elbow and shoulder joint arthropathy, vertebral spondylosis, and ossifying pachymeningitis. The musculoskeletal changes may be a reflection of long-lasting exposure to growth-promoting factors like GH and IGF-1 but can also be considered concurrent coincidental findings without a direct relation to pituitary somatotroph hyperfunction. However, various bone changes have also been documented in previous reports. In one case, CT revealed diffuse cortical thickening of multiple limb bones (27). Another case described extensive vertebral changes, with the entire spinal column presenting as a rigid, inflexible structure due to the presence of multiple fused osteophytes (25). Additionally, thickening of the skull has been reported in affected cats (31). In humans the most prevalent symptoms are musculoskeletal pain, arthropathy, carpal tunnel syndrome, proximal myopathy and fibromyalgia (32). Although in this case no specific bone-related clinical signs were reported, the exercise intolerance that was observed could potentially have been a consequence of pain or discomfort of the skeletal changes.

## 4 Conclusion

This is the second case report on a dog diagnosed with a pituitary somatotroph adenoma and concurrent DM that underwent transsphenoidal hypophysectomy. Elevated GH levels normalized within hours after surgery with simultaneous restoration of hyperinsulinemia to near normal levels. However, despite remission of HS within 1 week after hypophysectomy, the dog remained diabetic. It appears that insulin resistant DM in this dog with pituitary somatotroph hyperfunction, is much more refractory than reported in acromegalic cats after hypophysectomy.

## Data Availability

The original contributions presented in the study are included in the article/Supplementary material, further inquiries can be directed to the corresponding author.

## Supplementary material

SUPPLEMENTARY FIGURE S1 Photograph illustrating a 10-year-5-month male neutered Staffordshire bull terrier with acromegaly.

SUPPLEMENTARY FIGURE S2 Overview of postoperative glycemic control. The left panel shows blood glucose levels measured 4–6 hours after insulin administration, with up to two values per day. The right panel depicts insulin type and dosage (IU). Day 0 denotes the day of transsphenoidal hypophysectomy.
