# Supplementary figures and images for: Case Report: Treatment of hypersomatotropism in a diabetic dog with transsphenoidal hypophysectomy
Source: Front Vet Sci. 2026 Feb 24;13:1740713. doi: 10.3389/fvets.2026.1740713 (PMC12976564; doi:10.3389/fvets.2026.1740713)

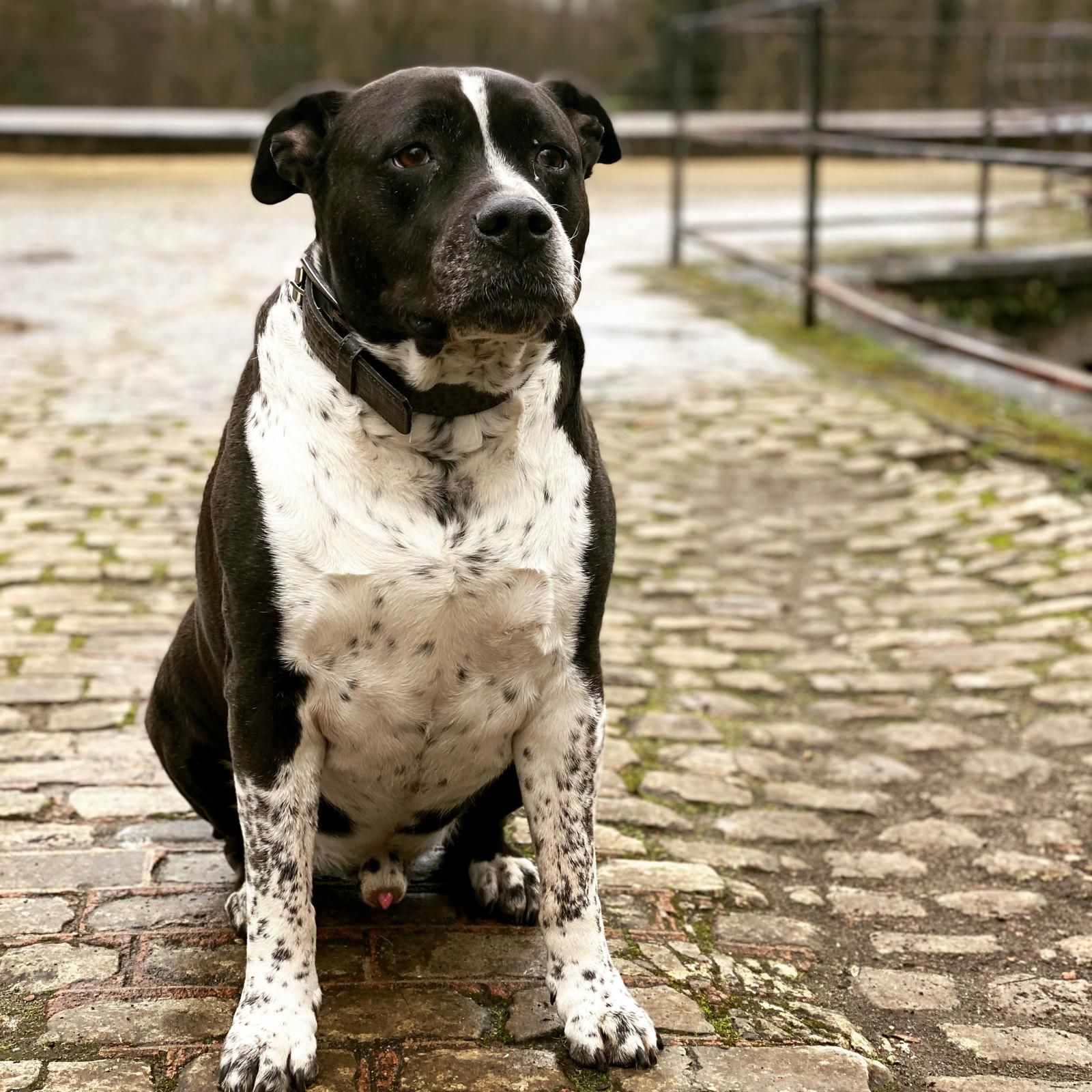

Supplement: SUPPLEMENTARY FIGURE S1 — Photograph illustrating a 10-year-5-month male neutered Staffordshire bull terrier with acromegaly. [file Image_1.JPEG]

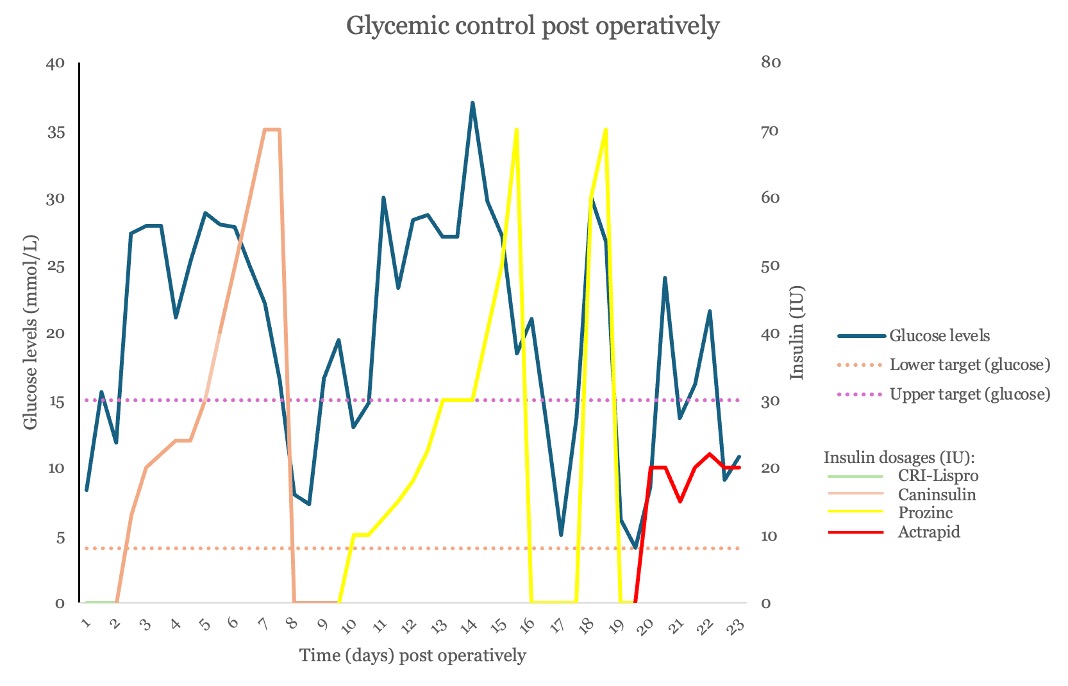

Supplement: SUPPLEMENTARY FIGURE S2 — Overview of postoperative glycemic control. The left panel shows blood glucose levels measured 4–6 hours after insulin administration, with up to two values per day. The right panel depicts insulin type and dosage (IU). Day 0 denotes the day of transsphenoidal hypophysectomy. [file Image_2.JPEG]
